# Comparative analysis of malt quality and starch characteristics of three South Korean barley cultivars

**DOI:** 10.1007/s10068-023-01419-6

**Published:** 2023-09-05

**Authors:** Jiyoung Park, Hyun-Jung Chung, Hye Young Park, Hyun-Jin Park, Sea-Kwan Oh

**Affiliations:** 1 Department of Central Area Crop Science, National Institute of Crop Science (NICS), Rural Development Administration (RDA), 126 Suin-ro, Kwonseon-gu, Suwon, Gyeonggi 16429 Republic of Korea; 2 Division of Food and Nutrition, Chonnam National University, Gwangju, 61186 Republic of Korea; 3 Department of Biotechnology, College of Life Sciences and Biotechnology, Korea University, 145 Anam-ro, Seongbuk-gu, Seoul, 02841 Republic of Korea; 4 National Institute of Crop Science (NICS), Rural Development Administration (RDA), 251 Chungyel-ro, Chuncheon, Gangwon 24219 Republic of Korea

**Keywords:** Malting, Beer barley, Malt quality, Barley starch, Malt starch

## Abstract

In this study, malt was produced in pilot-scale facilities and conditioned using three barley (*Hordeum vulgare* L.) cultivars in South Korea (Heugho, Hopum, and Kwangmaeg). Quality and starch characteristics were compared. The starch content was considerably reduced in all malts. Coleoptile elongation was higher in Heugho (HHM; 85.7% ± 12.6%) and Hopum (HPM; 83.9% ± 10.7%) than in Kwangmaeg (KMM; 78.1% ± 9.9%) malt. Malt yield ranged from 81.8 to 84.9%, with no significant difference. All samples presented type A crystallinity, and granules showed discoid shapes. After malting, the mono- and di-saccharide contents (not including sucrose) were increased. The fermentable sugar level was the highest in HHM, whereas non-fermentable sugar was the highest in KMM. These results suggest that HPM enables efficient scarification based on the rapid degradation of starch, while Heugho barley and HHM have a high potential for beer and malt production, respectively.

**Supplementary Information:**

The online version contains supplementary material available at 10.1007/s10068-023-01419-6.

## Introduction

Barley (*Hordeum vulgare* L., termed HVL) is one of the most important ingredients in beer brewing (Gupta et al., 2010). Malting is a critical step in beer production, alongside mashing and fermentation (Kok et al., 2019). It is a form of controlled grain germination that stabilizes barley seeds, leading to physical and chemical changes. Three essential steps are involved in the malting process. The first step is steeping, a process vital in ensuring adequate water absorption. The second is germination, whereby the embryo grows, enzymes are synthesized, and albumen decomposition is restricted. The third involves kilning for product stabilization (Ogushi et al., 2002). Starch, which constitute 51–77% of barley, is hydrolyzed to produce fermentable sugar, thus making it one of the most important factors in determining beer quality. Starch undergoes sequential modification during the malting, mashing, and fermentation processes of brewing (Fangel et al., 2018). Starch deformation begins during the malting process, and information on the structural change of starch is desirable for selecting suitable barley cultivars and improving the process (Wenwen et al., 2019). The quality of barley is analyzed and predicted from its pasting and thermal properties, which are related to the starch characteristics, including its molecular structure (Cozzolino, 2016). The structure of barley starch changes during the malting process owing to the large starch granule hydrolysis patterns, starch hydrolysis rate according to granule size, and easy hydrolysis of amylopectin, especially short-chain amylopectin (De Schepper et al., 2020). Additionally, starch molecular structure affects brewing, and amylose has a negative correlation with the degree of fermentation (Patindol et al., 2012). Starch structure analysis reveals whether malting has been performed adequately, which is an important step in improving brewing quality (Wenwen et al., 2019). Although analysis of the molecular structure of barley malt starch is essential for identifying and improving beer quality, no sufficient studies have been conducted in Korea to date.

The main ingredients used to brew beer, including hops, yeast, and barley, are mostly imported to South Korea. The proportion of domestic HVL ingredients is only 5%. In 2003, Hopum barley (HPB) was developed in South Korea as a novel, high-quality, and high-yield HVL cultivar with improved resistance to viral diseases and low protein content. HPB is the most frequently cultivated and used cultivar in Korea. Heugho barley (HHB), with a black-colored aleurone layer containing polyphenols and anthocyanins, was developed in 2014. It has attracted considerable interest from the industry as a source material for beer industries owing to the increased consumer demand for distinguished products with health benefits. Kwangmaeg barley (KMB) was developed in 2011 to improve adaptation to cold weather and allow cultivation in northern regions of the country (Kang et al., 2017). Although various barley cultivars have been developed in Korea, few malts, such as HPB, are commercially used. Moreover, little research has been conducted on the characteristics of newly developed barley cultivars or barley starch.

Therefore, we aimed to evaluate the malt quality and starch properties of Korean barley cultivars after pilot-scale malting to identify their brewing potential. In this study, the most common domestic HVL cultivar, HPB, and the two recently developed cultivars (HHB and KMB) were used in pilot-scale malting. The produced malts were analyzed in terms of proximate composition, enzymatic activity, elongation, and yield. Additionally, starch was isolated to investigate the starch structure and characteristics of barley and malt in each cultivar.

## Materials and methods

### Materials

**Fig. 1:**
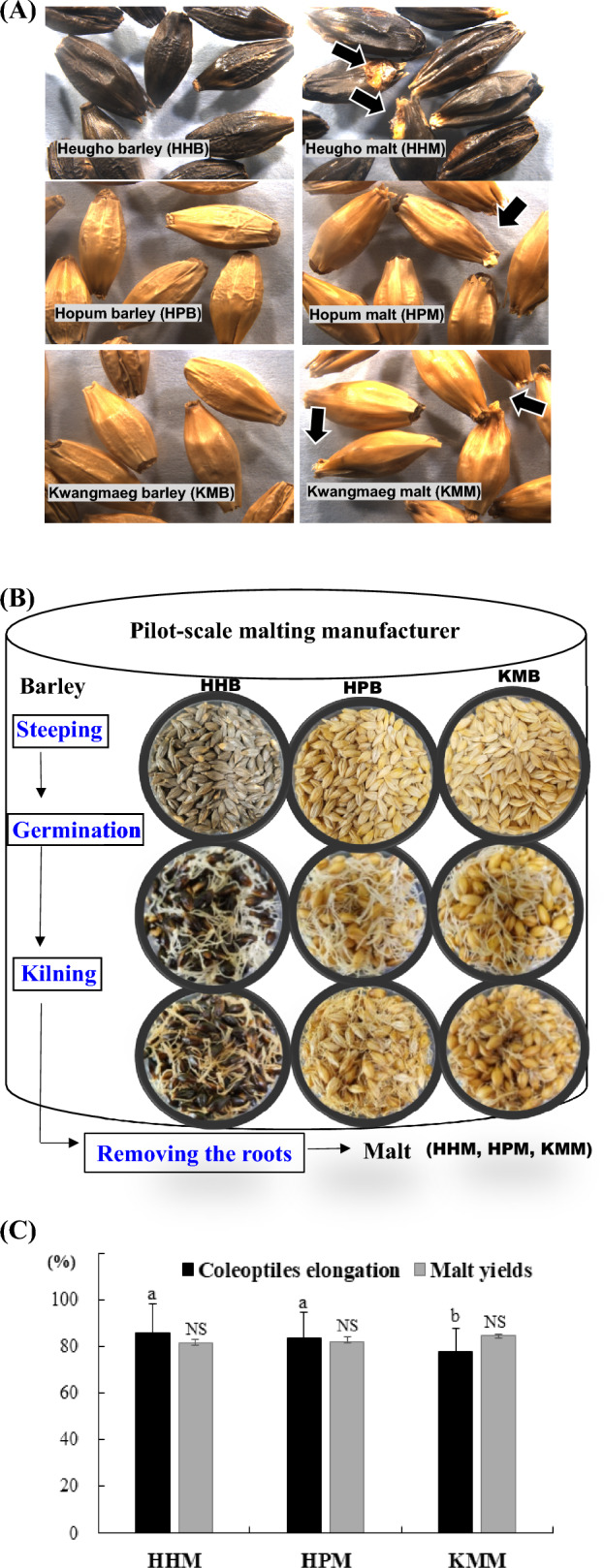
Appearance of barley and malt. (**A**) Malt (HHM, HPM, and KMM); the coleoptiles are indicated by arrows; (**B**) A scheme of pilot-scale malting process; (**C**) Coleoptile elongation (%) and malt yield. Y-axis, coleoptile elongation (%) and malt yield; a, b Values are statistically significant (P < 0.05) as determined via Duncan’s multiple range test; *NS* not statistically significant, *HHB* Heugho barley, *HPB* Hopum barley, *KMB* Kwangmaeg barley, *HHM* Heugho malt, *HPM* Hopum malt, *KMM* Kwangmaeg malt

Three two-row barley (*H. vulgare*) cultivars, developed for beer production in Korea, were used in this study: HHB, HPB, and KMB. The photographs are shown in Fig. 1A. The three cultivars were seeded in October 2019 and harvested in May 2020 in the Jeonju region. The cultivars were grown by the Rural Development Administration using standard cultivation procedures.

### Pilot-scale malting and sample preparation

The Department of Central Area Crop Science of the National Institute of Crop Science in Suwon, South Korea, has recently installed facilities (Dsntech Co. Hwasung, Korea) for pilot-scale malting (10–50 kg). For malting, the methods employed by Kim and Kang (2002) were used with modifications; the three cultivars of barley were treated equally, as presented in Fig. 1B. With a malting machine at the National Institute of Crop Science, 10 kg of malting was performed via steeping, germination, and kilning. Undamaged barley seeds that were not filtered through the 2.5-mm sieve (Doori Innovarion Corp. Gimje, Korea) were selected and washed. To steep barley with a moisture content range of 42%, we maintained the process at 18 °C for 50 h, followed by germination at 17 °C for 100 h; kilning was performed at 45 °C for 14 h and 75 °C for 7 h, followed by curing at 80 °C before termination. The dried malt was cooled to ≤ 35 °C, and the roots were removed for subsequent composition analysis and starch extraction (Park et al., 2022).

The malt was ground using a grinder (CT293 CyclotecTM, FOSS Analytical Co. Ltd., Suzhou, China). The flour, filtered using a 100-mesh standard sieve (No. 100 ASTM E11, Standard Test Sieve Scientific Co., Ltd., Wonju, Korea), was used in the analyses. The starch was isolated using the alkaline steeping method (El Halal et al., 2019). After removing water from the barley and malt that had been soaked in water for 4 h, 0.2% NaOH solution was added, and the barley and malt were crushed with a mixer and then passed through a 100-mesh sieve. Subsequently, a 0.2% NaOH solution was re-added and washed five times. Post supernatant removal, neutralization at a pH of 7.0 to 7.5 using 1N HCl, centrifugation at 2000×*g* for 20 min, and washing thrice with distilled water were conducted. After drying at 40 °C, the starch was ground in the same way as malt.

### Coleoptile elongation and malt yields

Coleoptile elongation and malt yield were measured following the methods of Kim and Kang (2002) and Oh et al. (2003), respectively, with some modifications. The ratio of the coleoptile length grown to the length of the seed was calculated, and coleoptile elongation was expressed as elongation rate (%). The average of 100 grains of malt measured using a digimatic caliper (CD-10APX, Mitutoyo Corporation, Kawasaki, Japan) was used for analysis. The malt yield was calculated as follows:

$${\text{Yield }}\left( \% \right)\, = \,\left( {{\text{malt weight }}\left( {\text{dry basis}} \right)/{\text{barley weight }}\left( {\text{dry basis}} \right)} \right)\, \times \,{1}00$$

### Composition analysis and enzyme activities of barley and malt flours

Moisture, crude ash, crude protein, and crude fat contents were analyzed following the methods described by Park et al. (2020). Briefly, moisture content was measured after incubation at 105 °C for 2 h. Crude ash was determined after incubation at 600 °C for 5 h. Crude protein was measured using the semi-micro-Kjeldahl method (Kjeltec 2400 AUT; Foss Tecator, Hilleroed, Denmark). Crude fat content was estimated via the Soxhlet extraction method using the Soxtec System HT 1043 extraction unit (Foss Tecator). A total starch assay kit, an amylose/amylopectin assay kit, an *α*-amylase SD assay kit, and a *β*-amylase assay kit, all obtained from Megazyme (Megazyme International, Ltd., Wicklow, Ireland), were used to determine the total starch and amylose content enzyme activities.

### Microscopic observation of barley and malt starch

Barley and malt starch samples were gold-coated to increase their conductivity and ease evaluation of their particle size under a scanning electron microscope (SEM-3000; Hitachi Ltd., Tokyo, Japan) at ×1000 and ×4000 magnifications.

### X-ray diffractometry

The crystalline structures of the starches were analyzed using an X-ray diffractometer (PANalytical, X’pert MPD high-resolution XRD, Almelo, Netherlands). The diffractometer was operated at 40 kV and 40 mA, with a scanning range of 5°–40°(2θ) and a scan rate of 2.0°/min. Starch crystallinity was quantitatively calculated according to the method described by López-Rubio et al. (2008) using the peak-fitting software (Origin 6.0, Microcal, Northampton, MA, USA).

### Molecular weight distribution

The molecular weights of barley and malt starch were analyzed using high-performance size exclusion chromatography (HPSEC). HPSEC (HELLOS, Wyatt Technology Co., Santa Barbara, CA, USA) was used with a multi-angle laser light scattering (MALLS) and refractive index (RI) detector. The starch samples (1.0 g) supplemented with 1–2 drops of ethanol were prepared in 100 mL of 90% dimethyl sulfoxide (DMSO) for 1 h at 100 °C with minor modification of the methodology employed by Han and Lim (2004). The solubilized starch molecules were precipitated by adding ethanol (500 mL), centrifuged, and dried. Solubilized starch (0.5%) was prepared in a 0.1 M NaOH buffer by heating the solution for 30 min at 95 °C. The solution was then neutralized with HCl (pH 7), autoclaved for 10 min, and filtered using a 0.45-µm syringe filter. The prepared sample was injected into the HPSEC analyzer fitted with an RI (Waters 2414, Waters Co., Milford, MA, USA) and MALLS detector. The separation was achieved using SEC columns (TSK G5000 PW, 7.5 mm × 600, TosoBiosep, Montgomeryville, PA, USA). The mobile phase was 0.15 M NaNO_3_ and 0.02% NAN3 (HPLC grade, flow rate: 0.4 mL min^−1^).

### Chain length distribution of amylopectin

The amylopectin chain length distributions of barley and malt starches were determined via a method previously described by Jeong et al. (2021).

Starches of barley and malt (10 mg) were dispersed in 2 mL of 90% DMSO and boiled with continuous stirring for 20 min. The dispersion was mixed with 6 mL absolute ethanol and centrifuged at 2000×*g* for 12 min. The precipitate was dissolved in 2 mL of 50 mM sodium acetate buffer (pH 3.5) and heated in a boiling water bath with continuous stirring for 20 min. After the solution was equilibrated to 37 °C, isoamylase (E-ISAMY, 4.16 µL, 240 U mg^−1^, Megazyme Co., Wicklow, Ireland) was added, and the starch solution was incubated at 37 °C with constant stirring at 150 rpm for 24 h. The sample was then boiled for 10 min for enzyme inactivation. An aliquot (200 μL) of the debranched starch was diluted with 2 mL of NaOH (150 mM). The sample was filtered using a 0.45-µm nylon syringe filter and injected into the High-Performance Anion-Exchange Chromatography with Pulsed Amperometric Detection (HPAEC-PAD) on a Dionex ICS-5000 system fitted with a CarboPac PA200 column (3 × 250 mm). Separation was achieved using a gradient eluent with 150 mM NaOH and 0.6 M sodium acetate in 150 mM NaOH at a flow rate of 0.5 mL min^−1^.

### Composition analysis of monosaccharides and oligosaccharides

The monosaccharides of barley and malt starch in Korean barley cultivars were determined as follows: the sample (4 mg) was stirred without heat treatment for 30 min in 2 mL of distilled water. After stirring, the supernatant was centrifuged (2000×*g*, 10 min, 4 °C) to achieve separation. The separated supernatant was filtered using a 0.45-μm micro filter and injected (10.0 μL) into the HPAEC-PAD on a Dionex ICS-5000 system (Thermo Fisher Scientific Inc., Waltham, MA, USA) fitted with a Carbopac PA 1 (4.0 × 250 mm). The mobile phase was 150 mM NaOH (isocratic) at a flow rate of 1.0 mL min^−1^.

The oligosaccharide content of barley and malt starch in Korean barley cultivars was analyzed in the same way as monosaccharides. Separation was achieved using a gradient eluent with 500 mM NaOAc in 100 mM NaOH and 100 mM NaOH at a flow rate of 1.0 mL min^−1^.

In this study, the total sugar content was determined by analyzing the sum of monosaccharides and oligosaccharides. Additionally, the fermentable sugar content was calculated by summing up glucose, maltose, and maltotriose content.

### Statistical analysis

All data are presented as means of triplicate measurements and analyzed using SAS v. 9.4 (SAS Institute Inc., Cary, NC, USA). Statistical significance was determined using one-way analysis of variance and Duncan’s multiple comparison test. P < 0.05 indicates statistically significant differences between treatment means.

## Results and discussion

### Barley and malt morphology, coleoptile elongation, and malt yield

The results of the microscopic examination of the morphology of the three Korean barley cultivars and the malt produced using the pilot-scale malting machine are presented in Fig. 1B. The seed coat color of HHB and HHM was black, whereas that of HPB, HPM, KMB, and KMM was golden. The seeds appeared uniform in size. After the removal of the root from all the samples (Fig. 1A), the size of the seeds did not appear to change significantly during malting, as observed visually; however, for all malts (HHM, HPM, and KMM), the coleoptiles were visible.

Malt coleoptile elongation and yield of the three Korean barley cultivars are shown in Fig. 1C. The coleoptile elongations for HHM, HPM, and KMM were 85.7% ± 12.6%, 83.9% ± 10.7%, and 78.1% ± 9.9%, respectively; HHM and HPM showed significantly higher coleoptile elongation than KMM (*P* < 0.05). The malt yield ranged between 81.8 and 84.9% (KMM > HPM > HHM), but no significant variation was found.

The barley coleoptiles appeared on the second day as they sprouted out under the husk. The growth rate was the fastest on the third day, and growth continued until the fifth day, on which coleoptile elongation has been reported to reach approximately 90% of barley seed length (Gibeaut et al., 2005). Farzaneh et al. (2017) showed that the malt yield decreased as the germination time increased, which was attributed to cellular respiration in the seed and the consequent decrease in starch content. In a study using Pilsner-type barley, the malt loss per barley cultivar by germination time ranged between 10.6 and 15.7%, and 4 days of germination are considered adequate to attain a suitable level of malt extraction and prolonged germination, up to the fifth or sixth day. This process has neither negative nor positive effects on malt characteristics (Zembold-Guła et al., 2009). Thus, the malt coleoptile elongation and yield of the three Korean barley cultivars, following malting under the same conditions as the pilot scale, were adequate. The malt was suitable for use as the sample in subsequent analyses.

### Malt flour composition analysis

The results of the composition analysis of the three Korean barley cultivars and the malt flour for moisture, ash, crude fat, crude protein, total starch, and amylose contents are presented in Table 1. In a previous study, total barley seeds showed 65–68% starch, 10–17% proteins, 2–3% crude fat, and 1.5–2.5% minerals (Gupta et al., 2010), which is consistent with our results. However, KMB and KMM had a lower starch content than that previously reported. In another study, the analysis of more than 50 varieties of beer barley in Korea showed that the average protein content was 12.3%. High protein content in barley was reported to have a negative correlation with malt quality. In this study, the protein content was lower than that in previous studies, and HHM showed the lowest value among them (Oh et al., 2003). Quek et al. (2019) reported that the starch content ranged between 53 and 62% depending on the germination time, and the barley cultivar was reduced to the range of 3–6% after malting, whereas the crude protein content remained similar or increased to 7–11% (Quek et al., 2019). The crude protein content showed a notably steep increase in the early stage of germination, which was attributed to the rapid early reduction in the non-protein dry mass during the initial 24 h of germination or the utilization of carbohydrates or lipids for protein production. In this study, the starch content after malting notably decreased to 1.4–8% (HPM > KMM > HHM). The crude protein content decreased by 1% except for KMM, which showed the lowest level of coleoptile elongation, suggesting that germination progressed adequately. Pinkaew et al. (2017) claimed that the decrease in starch content was attributed to the conversion of a certain proportion of starch into sugar moieties as the starch hydrolases, such as α amylase and β-amylase, were activated during germination. In this study, HPM and KMM with high α-amylase activity had a greater reduction in starch content than HHM.

**Table 1:** Composition, amylose content, and enzyme activities in Korean barley and malt starch varieties

Samples	Composition						Enzyme activities	
	Moisture (%)	Ash (%)	Crude fat (%)	Crude protein (%)	Total starch (%)	Amylose (%)	α-amylase	β-amylase
							(Amylase SD units/g)	
HHB	11.0 ± 0.0^a^	1.8 ± 0.0^b^	2.4 ± 0.2^a,b^	10.4 ± 1.3^b^	66.6 ± 0.4^b^	24.4 ± 0.3^c^	0.2 ± 0.0^d^	28.6 ± 0.6^b,c^
HHM	8.4 ± 0.0^b^	1.6 ± 0.1^d^	2.2 ± 0.1^b^	9.3 ± 0.1^c^	65.2 ± 0.8^c^	25.9 ± 0.1^a^	140.3 ± 3.9^c^	20.6 ± 0.2^d^
HPB	11.1 ± 0.1^a^	1.9 ± 0.0^b^	2.5 ± 0.1^a,b^	11.3 ± 0.3^a,b^	67.9 ± 0.5^a^	25.0 ± 0.1^b^	0.6 ± 0.1^d^	27.6 ± 0.2^c^
HPM	7.4 ± 0.2^c^	1.7 ± 0.1^c^	2.3 ± 0.1^a,b^	10.3 ± 0.1^b,c^	59.9 ± 0.4^e^	23.9 ± 0.2^d^	174.9 ± 2.3^b^	19.3 ± 0.1^d^
KMB	10.8 ± 0.1^a^	1.8 ± 0.1^b,c^	2.6 ± 0.1^a^	11.1 ± 0.5^a,b^	61.7 ± 0.5^d^	23.6 ± 0.5^d^	0.3 ± 0.2^d^	52.4 ± 3.0^a^
KMM	7.3 ± 0.2^c^	2.2 ± 0.1^a^	2.5 ± 0.2^a,b^	11.9 ± 0.1^a^	56.1 ± 0.3^f^	21.6 ± 0.2^e^	180.4 ± 1.5^a^	30.3 ± 0.6^b^

Values are presented as means ± SD of three independent trials

*HHB* Heugho barley, *HPB* Hopum barley, *KMB* Kwangmaeg barley, *HHM* Heugho malt, *HPM* Hopum malt, *KMM* Kwangmaeg malt

^a–f^Values with different letters within a column are significantly different (P < 0.05), as determined by using Duncan’s multiple range test

The amylose content in the three Korean barley cultivars and malt flour ranged from 21.6 to 25.9%, all of which indicated normal barley cultivars. Among the three cultivars, KMB had the lowest amylose content (23.6%). After malting, the amylose content in the HHM cultivar increased, whereas that in the HPM and KMM decreased.

According to a previous study, the starch content decreased during the process of malting, whereas the amylose content increased in the early stage, which was attributed to the α-amylolysis of the amylose and amylopectin constituting the starch (Arendt and Zannini, 2013). Park et al. (2020) reported that the amylose content increased in high-amylose rice starch following hydrolysis mediated by α-amylase, whereas the measured content varied according to the analytical method (Con A vs. iodine binding). This finding suggests that malting results in varying patterns of starch hydrolysis, for which further studies should be conducted, especially regarding molecular structures.

### Starch hydrolase activity

The results of the activities of starch hydrolases, α-amylase, and β-amylase, of the three Korean barley cultivars and the malt flour are presented in Table 1.

The α-amylase activity was negligible at 0.2–0.6 units g^−1^ for the barley samples. However, it was 140.3–180.4 units g^−1^ for the malt flour samples (KMM > HPM > HHM). In contrast, the β-amylase activity was high at 27.6–52.4 units g^−1^ for the barley samples and was reduced in all cultivars after malting. Steeping and germination of barley seeds facilitated starch hydrolase production, as demanded by the conversion of starch into oligosaccharides. Moreover, with the increase in germination time from three to seven days, the α-amylase activity was high, as it reached the maximum level on the sixth day and began to decrease from the seventh day (Farzaneh et al., 2017). Our study also found an increase in α-amylase activity after malting, which is consistent with previous research. The variation in enzyme activity in malt flours produced under identical conditions is presumably owing to the unique cultivar characteristics. The β-amylase activity decreases at 55 °C, and the malting processes affect the variation in enzyme activity between barley and malt flour (Quek et al., 2019). The authors also reported a negative correlation between α-amylase activity and total starch content during germination (Pinkaew et al., 2017). However, β-amylase activity did not show a remarkable change (Quek et al., 2019). Similarly, in the present study, the total starch content was detected in the following order: HHM > HPM > KMM, and α-amylase activity was detected in the following order: KMM > HPM > HHM, with remarkable differences that are consistent with previous studies. Collectively, these findings indicate that not only high α-amylase activity but also a high level of total starch content should be considered to ensure efficient alcohol production.

### Morphology

**Fig. 2:**
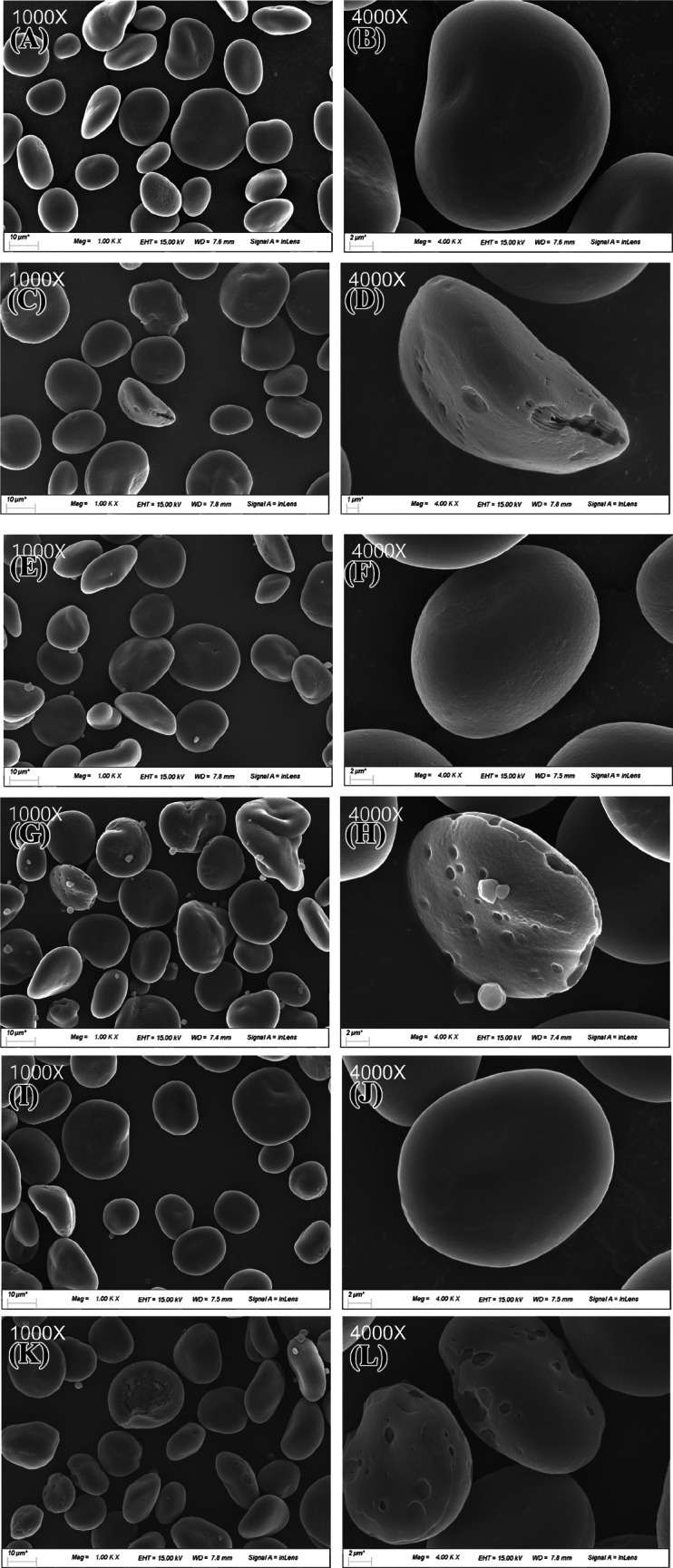
Scanning electron micrographs of barley and malt starches in Korean varieties at ×1000 (**A**, **C**, **E**, **G**, **I**, **K**) and ×4000 magnification (**B**, **D**, **F**, **H**, **J**, **L**). (**A**) HHB, (**B**) HHB, (**C**) HHM, (**D**) HHM, (**E**) HPB, (**F**) HPB, (**G**) HPM, (**H**) HPM, (**I**) KMB, (**J**) KMB, (**K**) KMM, and (**L**) KMM. *HHB* Heugho barley, *HPB* Hopum barley, *KMB* Kwangmaeg barley, *HHM* Heugho malt, *HPM* Hopum malt, *KMM* Kwangmaeg malt

Scanning electron microscope images of the morphology of the three Korean barley cultivars and starch isolated from the malt are presented in Fig. 2 at ×1000 and ×4000 magnification, respectively. The barley starch was discoid-shaped. The observed oblong shapes of the starch grains varied in size; most were approximately 10 μm, but some were as large as 20–30 μm. The starch grains of all barley samples showed smooth surfaces (Fig. 2A, B, E, F, I, J), whereas the malt samples showed rough surfaces (Fig. 2C, D, G, H) with marks indicating enzyme hydrolysis. Particularly, HHM showed a trace of a long furrow (Fig. 2D). HPM showed broken starch grains as small as 1–2 μm particles (Fig. 2H).

In a previous study, the starch morphology observed for malt was reported to vary according to the starch particle size owing to varying patterns of α-amylolysis (Myllrinen et al., 1998). Wenwen et al. (2019) observed pinholes in large starch grains as the hydrolysis proceeded from the interior to the exterior and suggested that the traces of rough furrows on the exterior without pinholes for small starch grains reflected corresponding hydrolysis patterns (Wenwen et al., 2019).

In this study, the size of the starch was generally similar across the three barley cultivars and malt flour, but the starch morphology did not appear identical. HHM showed traces of deep furrows owing to hydrolysis from the interior, along with pinholes. In contrast, HPM and KMM, despite having varying diameters and depths, showed an overall shallow and spherical holes owing to hydrolysis on the exterior. Moreover, HPM exhibited numerous small starch particles, indicating a higher level of fragility than other malts. As the effects on starch hydrolysis and glycosylation vary, further studies should be conducted.

### X-ray diffractometry

**Fig. 3:**
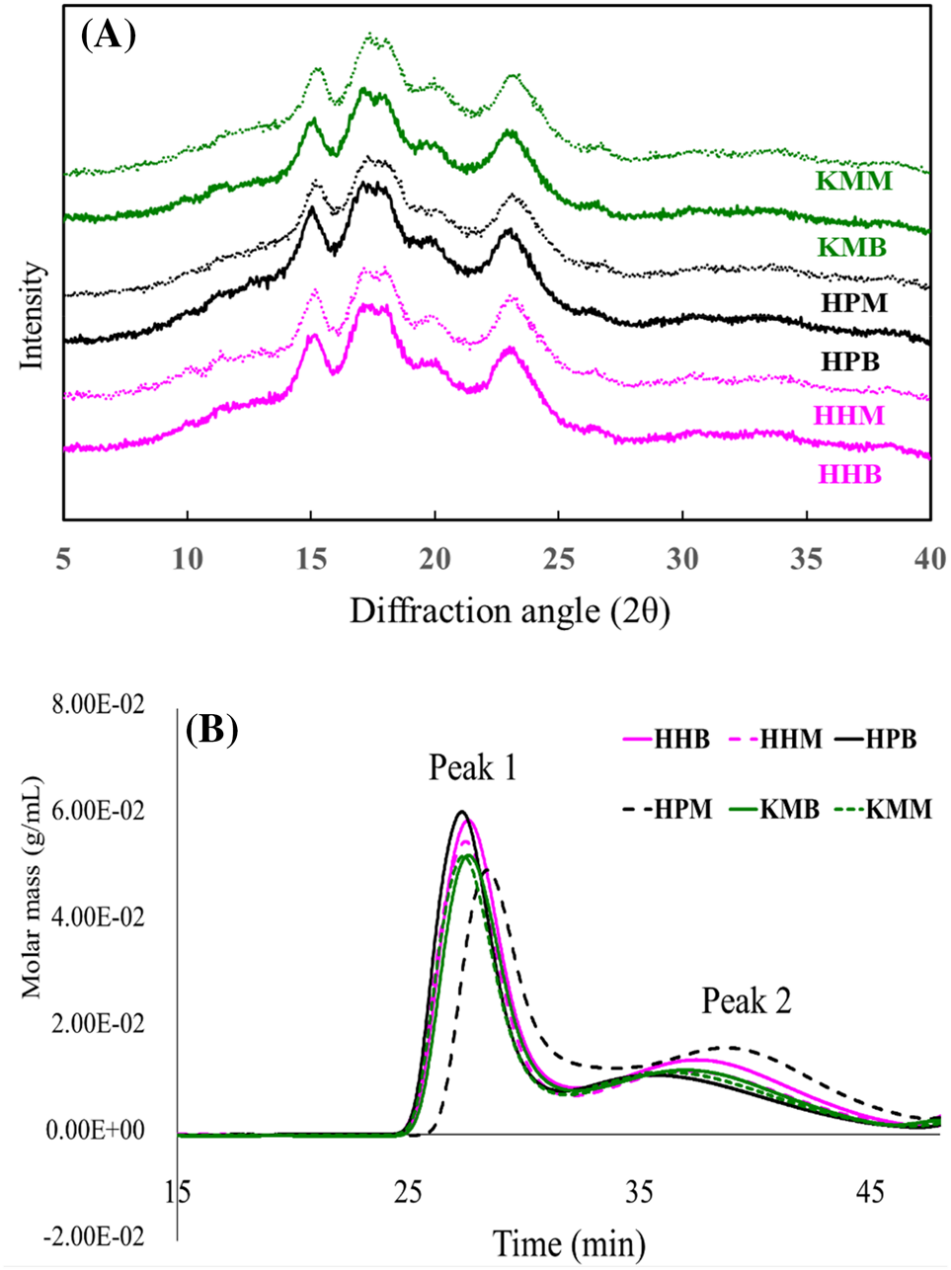
Crystallinity and chromatograms of starch isolated from the three Korean barley cultivars and malt flour. (**A**) X-ray diffraction patterns of barley and malt starches from Korean barley cultivars. (**B**) High-performance size exclusion chromatography (HPSEC) chromatogram of malt starch from different cultivars; Peak 1 (amylopectin), Peak 2 (amylose). *HHB* Heugho barley, *HPB* Hopum barley, *KMB* Kwangmaeg barley, *HHM* Heugho malt, *HPM* Hopum malt, *KMM* Kwangmaeg malt

The crystallinity results based on X-ray diffractometry for starch isolated from the three Korean barley cultivars and malt flour are presented in Fig. 3A. The relative crystallinities are listed in Table 2. For all barley cultivars and malt samples, two distinct peaks at 15° 2θ and 17° 2θ, 18° 2θ, and a clear peak at 23° 2θ were observed to indicate a typical A-type starch structure (Fig. 3A). No changes in crystallinity occurred during malting. This result is consistent with that of a previous study in which barley starch showed no structural change during the malting process, where malting occurred mainly in the amorphous region and not in the crystalline region (Contreras-Jiménez et al., 2019). In another study, the malting of sorghum seeds did not change the crystal structure of starch, and no interaction between starch hydrolases and crystal structure occurred (Oseguera-Toledo et al., 2020). A recent study using corn in malting observed fine holes in the starch particles in the regions of hydrolase attack and no change of A-type starch crystal structures as the enzymatic activity was selective during malting. The results indicated that an enzymatic attack had occurred in the amorphous region of the starch particles (Hernández-Becerra et al., 2020).

**Table 2:** Relative crystallinity, molecular weight, and amylopectin chain length distribution of Korean barley and malt varieties

Samples	Relative crystallinity (%)	Peak I		Peak II		Chain length distribution (%)				Average chain length
		Mw (× 10^6^ g/mol)	Fraction mass (%)	Mw (× 10^6^ g/mol)	Fraction mass (%)	DP 6–12	DP 13–24	DP 25–36	DP ≥ 37	
HHB	21.7 ± 0.6^a,b^	211.4 ± 7.3^a^	62.6^b^	19.5 ± 1.1^b^	37.4^c^	25.6 ± 0.5^ns^	46.5 ± 0.4^a^	15.5 ± 0.0^c^	12.4 ± 0.2^ns^	21.4 ± 0.1^ns^
HHM	21.0 ± 0.8^b^	191.1 ± 6.4^b^	58.8^c^	11.8 ± 0.8^c^	41.5^b^	25.5 ± 0.6^ns^	46.2 ± 0.3^a,b^	15.7 ± 0.2^a,b^	12.5 ± 0.7^ns^	21.5 ± 0.3^ns^
HPB	18.5 ± 0.1^c^	220.6 ± 9.7^a^	65.1^a^	32.5 ± 1.5^a^	34.9^d^	25.5 ± 0.7^ns^	46.4 ± 0.2^a,b^	15.5 ± 0.1^b,c^	12.7 ± 0.7^ns^	21.5 ± 0.4^ns^
HPM	17.5 ± 0.3^d^	113.8 ± 7.5^d^	54.6^d^	4.7 ± 0.2^e^	45.4^a^	26.2 ± 0.6^ns^	46.4 ± 0.1^a,b^	15.2 ± 0.1^d^	12.3 ± 0.6^ns^	21.2 ± 0.3^ns^
KMB	22.4 ± 0.3^a^	188.9 ± 5.5^b^	60.0^c^	9.0 ± 0.2^d^	40.0^b^	26.1 ± 0.5^ns^	45.9 ± 0.4^a,b^	15.6 ± 0.1^b,c^	12.5 ± 0.8^ns^	21.4 ± 0.3^ns^
KMM	21.7 ± 0.6^a,b^	169.0 ± 4.0^c^	60.3^c^	8.7 ± 0.5^d^	39.7^b^	25.6 ± 0.5^ns^	45.8 ± 0.5^a,b^	15.8 ± 0.1^a^	12.8 ± 0.9^ns^	21.6 ± 0.4^ns^

Values are presented as means ± SD of three independent trials

*ns* not statistically significant, *DP* degree of polymerization, *HHB* Heugho barley, *HPB* Hopum barley, *KMB* Kwangmaeg barley, *HHM* Heugho malt, *HPM* Hopum malt, *KMM* Kwangmaeg malt

^a–e^Values with different letters within a column are significantly different (P < 0.05), as determined via Duncan’s multiple range test

The relative crystallinity was detected in the following order: KMB > HHB > HPB, with HPB exhibiting a significantly (P < 0.05) low level of 18.5% (Table 2). In line with previous studies (Hernández-Becerra et al., 2020), no change in A-type crystal structures was observed in this study; however, a peak of weak intensity was observed at 17° 2θ and 18° 2θ for HPM, and the relative crystallinity was reduced across all cultivars after malting (Table 2). This suggests that certain crystalline regions may experience a slight decrease due to the activity of starch hydrolase during malting.

Park et al. (2020) reported a substantial decrease in peak intensity in X-ray diffraction following rice starch hydrolysis using α-amylase and glucoamylase, as well as a significant decrease in the relative crystallinity, as demonstrated in this study. The variation in the intensity of crystallinity in this study was not high; the decrease in relative crystallinity for the malt starch was within 1%, implying a lack of significant influence of malting process on the crystalline region of barley starch.

The relative crystallinity (%) was estimated to determine its effects on starch structure at each step of malting. Until the third day of germination, the relative crystallinity increased because the enzymes attacked the amorphous region of starch. Crystallinity decreased from the fourth day of germination or kilning, and a possible association with amylopectin debranching has been suggested (Contreras-Jiménez et al., 2019).

### Molecular weight analysis and chain length distribution

The HPSEC chromatograms of starch isolated from the three Korean barley cultivars and malt flour are presented in Fig. 3B. The results of molecular weight analysis for peaks 1 and 2 are presented in Table 2. Peaks 1 and 2 indicate amylopectin and amylose, respectively. HPB and HHB displayed the highest amylopectin molecular weights, which were considerably higher than those of KMB. All samples showed a decrease in molecular weight after malting; HPB, in particular, showed the highest reduction to 2.2 × 10^6^ for peak 1 and 1.1 × 10^6^ for peak 2, implying the lowest molecular weight distribution. In contrast, HHB and KMB exhibited a decrease in molecular weight for peak 1 after malting. For HPB, the molecular weight of amylopectin was reduced, whereas that of amylose was increased. The peak 2 fraction mass (%) significantly increased in HHM and HPM (P < 0.05) but decreased in KMM (P > 0.05) compared with that in the respective barley samples. However, KMB and KMM showed the least variation in starch molecular weight during malting.

The amylopectin molecular chain distribution and mean chain length obtained from HPAEC-PAD are listed in Table 2. No significant variation was observed among the samples with a degree of polymerization (DP) 6–12 for the shortest chain length and DP > 37 for the longest chain length. However, a slight variation was found for the DP 13–36 for the intermediate chain length. DP 25–36 showed a notable increase in all samples except for HPB and HPM. The percentage of DP 6–12 showed a slight increase in HPM than in HPB, at a level higher than that in the other samples.

In this study, the amylopectin distribution and mean chain length before and after malting for each barley cultivar showed no significant differences. Nonetheless, the molecular weight distribution showed a considerable decrease in peak 1 (amylopectin), implying that the cause of the reduced molecular weight and starch crystallinity may be owing to amylopectin debranching rather than amylose content or chain length distribution during malting (Table 2). The attack of starch hydrolytic enzymes influenced the amorphous region (Hernández‐Becerra et al., 2020). The starch crystallinity was lower in HPB than in the other two cultivars. In addition, the molecular weight of amylopectin in HPB was the highest with HHB, and the fraction mass (%) was the highest with HPM. Nevertheless, after malting, HPM had the lowest molecular weight and fraction mass (%) among the cultivars. Similarly, the fraction mass (%) of amylose in HPB was the lowest; however, the amylose molecular weight was the lowest in HPM, and the fraction mass of amylose (%) was the highest. In Fig. 2G and H, we observed that the small or broken starch particles and the starch surface were greatly modified. Chu et al. (2014) reported that short branches of amylose hydrolyze faster than long branches during malting. This indicates that starch degradation in HPM occurred faster than in other cultivars (Chu et al., 2014).

### Sugar composition

The results of the analysis of monosaccharides and oligosaccharides in the starch isolated from the three Korean barley cultivars and malt flour are presented in Table 3. Mannitol, a type of sugar alcohol, was not detected in any of the barley samples tested. The mannitol content varied significantly across the malt samples, with HPM showing the highest content, followed by KMM and HHM. With the exception of sucrose, the contents of arabinose, glucose, fructose, and maltose increased for mono- and disaccharides in all samples after malting. For oligosaccharides, only maltotriose was detected, and none of the detected oligosaccharides were DP 4 or above. The malt samples exhibited a higher content of maltotriose than the barley samples. The sugar composition of the malt flour contained high percentages of maltose and glucose. HHM had the highest maltose and maltotriose contents among the fermentable sugars, while KMM had a markedly higher content of fructose, which is not fermented. In previous studies, barley particle size was reported to affect the sugar composition developed during saccharification. Larger starch particles lead to greater sugar production, and smaller particles lead to greater dextrin production (Langenaeken et al., 2019). Large starch particles were around 20 µm, and small particles were visible particles within 5 µm. In this study, the comparison of starch morphology revealed that the particle size ranges from 10 to 20 µm (Fig. 2), and the lack of oligosaccharides indicated that the barley cultivars had starch particles suitable for producing mono- and disaccharides that are required in beer fermentation. Fructan is a fructose oligomer comprising one or more unbound glucose molecules. This oligomer increases slightly during malting and can be hydrolyzed in the form of sucrose, fructose, or glucose by invertases (Magalhães et al., 2016; Weschke et al., 2003). Sucrose is the preferred sugar used as the energy source of the seed embryo and is produced during germination (Weschke et al., 2003). In contrast, maltotriose accounts for 20% of fermentable sugar in brewing wort, and its use is very important; maltose accounts for 60% of fermentable sugars, making it the most abundant fermentable sugar. Additionally, glucose, fructose, and sucrose account for approximately 20% of the total sugar (Magalhães et al., 2016). The barley used in this study had a relatively high fermentable sugar content; in particular, HHM and HPM contain more than 80% fermentable sugar, proving its potential for better brewing (Table 3). However, further research is needed to unravel its brewing characteristics.

**Table 3:** Sugar composition of barley and malt starch varieties in Korea

Samples	Monosaccharides (%)						Oligo-saccharide (%)	Total sugar	Fermentable sugar (FS, %)	Ratio of FS/TS (%)
	Mannitol	Arabinose	Glucose	Fructose	Sucrose	Maltose	Maltotriose (DP = 3)	(TS, %)		
HHB	ND^d^	0.03 ± 0.01^e^	0.62 ± 0.05^d^	1.92 ± 0.08^d^	3.91 ± 0.24^a^	4.33 ± 0.24^c^	0.03 ± 0.04^c^	10.85 ± 0.78^c^	4.98 ± 0.45^c^	45.95
HHM	1.23 ± 0.01^c^	0.29 ± 0.01^b^	7.10 ± 0.46^b^	3.49 ± 0.08^b^	1.89 ± 0.02^d^	29.93 ± 0.02^a^	3.98 ± 0.63^a^	47.92 ± 4.36^a^	41.01 ± 4.22^a^	85.60
HPB	ND	0.05 ± 0.00^d^	0.67 ± 0.07^d^	1.90 ± 0.11^d^	3.76 ± 0.23^a^	3.96 ± 0.23^c^	0.05 ± 0.03^c^	10.38 ± 0.84^c^	4.67 ± 0.49^c^	45.03
HPM	2.02 ± 0.02^a^	0.36 ± 0.00^a^	5.65 ± 0.40^c^	3.28 ± 0.03^c^	1.27 ± 0.04^e^	24.70 ± 0.04^b^	3.51 ± 0.33^a,b^	40.79 ± 2.07^b^	33.87 ± 1.98^b^	83.04
KMB	ND^d^	0.03 ± 0.00^e^	0.49 ± 0.05^d^	1.95 ± 0.08^d^	3.41 ± 0.08^b^	3.41 ± 0.08^c^	0.02 ± 0.39^c^	9.31 ± 0.41^d^	3.93 ± 0.26^d^	42.17
KMM	1.36 ± 0.02^b^	0.25 ± 0.00^c^	8.65 ± 0.43^a^	8.34 ± 0.24^a^	3.06 ± 0.10^c^	25.91 ± 0.10^b^	3.18 ± 0.87^b^	50.75 ± 3.80^a^	37.74 ± 3.43^a,b^	74.37

No other monosaccharides (rhamnose, mannose, ribose, and cellobiose) and oligosaccharide (DP ≥ 4), except maltotriose, were detected

Total Sugar the sum of monosaccharides and oligosaccharides, Fermentable sugar: the sum of glucose, maltose, and maltotriose. Ratio of FS/TS: the ratio of fermented sugars to total sugars

*HHB* Heugho barley, *HPB* Hopum barley, *KMB* Kwangmaeg barley, *HHM* Heugho malt, *HPM* Hopum malt, *KMM* Kwangmaeg malt

^a–e^Values with different letters within a column are significantly different (P < 0.05), as determined by using Duncan’s multiple range test. Values are presented as means ± SD of three independent trials

### Supplementary Information

Below is the link to the electronic supplementary material.

Supplementary file1 (DOCX 18 KB)
