# Supplementary material for: Comparative analysis of malt quality and starch characteristics of three South Korean barley cultivars
Source: Food Sci Biotechnol. 2023 Sep 5;33(5):1135–45. doi: 10.1007/s10068-023-01419-6 (PMC10908982; doi:10.1007/s10068-023-01419-6)
Supplement: Supplementary file 1 — Supplementary file1 (DOCX 18 KB) [file 10068_2023_1419_MOESM1_ESM.docx]

Comparative Analysis of Malt Quality and Starch Characteristics of Three South Korean Barley Cultivars

**Analysis of thermal properties**

The thermal properties of heat-treated BRF were determined using a differential scanning calorimeter (DSC) (DSC Q1000; TA Instruments Inc., New Castle, DE, USA). Nine milligrams of sample was accurately weighed, mixed with 21 mg of distilled water, and hermetically sealed in high volume pans (TA Instruments Inc., New Castle, DE, USA). The samples were equilibrated 24 h to 25°C, and the pan was heated from 20°C to 150°C at a rate of 10 °C/min. An empty aluminum pan served as a reference. Data were calculated using the endotherm plot in the DSC software.

**Thermal properties of Korean barley and malt varieties**

The gelatinization characteristics of barley and malt starch through DSC analysis are presented in Table S1. The onset gelatinization temperature was 56.8 to 59.3℃; the peak gelatinization temperature ranged from 60.6 to 62.7℃, and the enthalpy was in the range of 10.9 to 12.3 J/g. The enthalpy showed no statistical difference. The gelatinization temperature showed statistical differences between HPM>HHM, KMM>HHB>HPB, and KMB, and all gelatinization temperatures, such as onset, peak, and completion temperature, tended to show high malt.

**Table S1. Thermal properties of Korean barley and malt varieties**

|  | Gelatinization | | | |
| --- | --- | --- | --- | --- |
| Sample | To (℃) | Tp (℃) | Tc (℃) | ΔH (J/g) |
| HHB | 57.9±0.0^3^ | 61.2±0.1^3^ | 67.5±0.3^2^ | 11.8±0.5^ns^ |
| HHM | 58.8±0.1^2^ | 62.1±0.2^2^ | 68.7±0.4^1^ | 11.6±0.3 ^ns^ |
| HPB | 57.0±0.1^4^ | 60.9±0.2^3,4^ | 67.7±0.4^2^ | 12.3±0.6 ^ns^ |
| HPM | 59.3±0.0^1^ | 62.7±0.1^1^ | 69.1±0.2^1^ | 11.5±0.4 ^ns^ |
| KMB | 56.8±0.2^4^ | 60.6±0.2^4^ | 67.0±0.2^2^ | 11.8±0.3 ^ns^ |
| KMM | 58.6±0.3^2^ | 62.5±0.4^1,2^ | 69.5±0.4^1^ | 10.9±1.1 ^ns^ |

1–4 Values with different letters within a column are significantly different (P < 0.05), as determined by using Duncan's multiple range test. ns = not statistically significant. Values are presented as means ± SD of three independent trials. DP, degree of polymerization. HHB, Heugho barley; HPB, Hopum barley; KMB; Kwangmaeg barley; HHM, Heugho malt; HPM, Hopum malt; KMM; Kwangmaeg malt. To: Onset gelatinization temperature Tp: Peak gelatinization temperatures, Tc: completion gelatinization temperature, ΔH: enthalpy
